# Association between fractional amplitude of low-frequency fluctuation (fALFF) and facial emotion recognition ability in first-episode schizophrenia patients: a fMRI study

**DOI:** 10.1038/s41598-022-24258-7

**Published:** 2022-11-15

**Authors:** Qijie Kuang, Sumiao Zhou, Haijing Li, Lin Mi, Yingjun Zheng, Shenglin She

**Affiliations:** Department of Psychiatry, The Affiliated Brain Hospital of Guangzhou Medical University, Guangzhou, 510370 People’s Republic of China

**Keywords:** Diseases, Psychiatric disorders, Schizophrenia

## Abstract

It was still unclear that the correlation between the resting-state intrinsic activity in brain regions and facial emotion recognition (FER) ability in patients with first-episode schizophrenia (FSZ). Our aim was to analyse the correlation between the fractional amplitude of low-frequency fluctuation (fALFF) and FER ability in FSZ patients. A total of 28 patients with FSZ and 33 healthy controls (HCs) completed visual search tasks for FER ability. Regions of interest (ROIs) related to facial emotion were obtained from a previous meta-analysis. Pearson correlation analysis was performed to understand the correlation between fALFF and FER ability. Our results indicated that the patients performed worse than the HCs in the accuracy performances of happy FER and fearful FER. The previous meta-analysis results showed that the brain regions related to FER included the bilateral amygdala (AMY)/hippocampus (HIP), right fusiform gyrus (FFG), and right supplementary motor area (SMA). Partial correlation analysis showed that the fALFF of the right FFG was associated with high-load fearful FER accuracy (r = − 0.60, *p* = 0.004). Our study indicated that FER ability is correlated with resting-state intrinsic activity in brain regions related to facial emotion, which may provide a reference for the study of FER deficiency in schizophrenia.

## Introduction

Schizophrenia is characterized by various clinical symptoms, such as hallucinations, delusions, and cognitive deficiency^1^. In recent years, social cognition in schizophrenia has received increased attention. Social cognition refers to a variety of cognitive processes involved in the accurate perception of the dispositions and intentions of others^2^. Bora et al.^3^ conducted a meta-analysis and showed that social cognition deficiency was consistent among patients with schizophrenia (including acute and chronic phases) and persisted even after other clinical symptoms were alleviated, which suggested that they might constitute a marker of schizophrenia.

Facial emotion recognition (FER) deficiency is one of the impaired social cognition in schizophrenia^4,5^. Previous study have shown that deficiency in FER are stable across phases of schizophrenia and are already present in first-episode schizophrenia (FSZ) patients^6^. In addition, evidence suggests that FER ability impaired is not associated with psychotic symptoms^7^. She et al.^8^ found that the FER ability of patients with FSZ was impaired in a visual search task, while the visual search function of graphics showed no abnormalities, implying that facial perception deficiency may be a specific marker for schizophrenia. On the other hand, FER ability is an independent predictor of social cognitive outcome prognosis in schizophrenia^9,10^. It may be associated with social withdrawal and hypervigilance to social threat, which impairs social functioning^11^. Taken together, these investigations revealed that FER is one of the social cognitive impairment features of schizophrenia and contributes to poor social functioning.

To date, an extensive body of magnetic resonance imaging (MRI) research has been conducted to explore the changes in processing facial emotion in patients with schizophrenia. The results of structural MRI studies have shown that the grey matter volume (GMV) of the frontal (particularly the medial and inferior portions), temporal lobes and amygdala (AMY)/hippocampus (HIP) are related to the facial emotion cognitive function of patients with FSZ^12,13^. For functional MRI (fMRI), a meta-analysis demonstrated brain activity changes across widespread brain regions when processing facial expressions of emotions, including the AMY, the fusiform gyrus (FFG), the HIP, the superior, medial and dorsolateral frontal cortices and the subcortical structures, which were weaker in patients with schizophrenia than in HCs^14,15^. Notably, both the GMV and neural activity in the FFG were lower in schizophrenia patients than in HCs^16^, which is termed the fusiform face area (FFA)^17^.

Although numerous fMRI studies have examined brain activation during FER, few studies have examined the correlation between intrinsic brain activity (IBA) and FER in patients with schizophrenia. The fractional amplitude of low-frequency fluctuation (fALFF) is one method used to analyse intrinsic brain activity with less physiological noise effects^18,19^. Compare to function connectivity, fALFF can directly reflect the IBA levels in voxels according to their energy levels in the resting state. Recently, Luo et al. revealed the genetic-brain-cognition mediation pathway in patients with schizophrenia by the GMV and fALFF methods, suggesting that the fALFF can be used to represent the IBA and can be used for studying cognitive deficiency in schizophrenia^20^.

To date, few studies analysed the correlation between resting-state intrinsic activity in brain regions associated with facial emotion and FER ability in patients with FSZ. Therefore, we adopted the fALFF method and visual search task to measure brain region intrinsic activity and FER ability. We hypothesized that the fALFF in brain regions related to facial emotion would be related to FER ability in patients with schizophrenia.

## Methods

### Subjects

FSZ patients were selected based on the following inclusion criteria: (1) Must have normal or corrected-to-normal vision; (2) Completely satisfied the DSM-IV (Diagnostic and Statistical Manual of Mental Disorders, Fourth Edition) criteria for diagnosis of schizophrenia, wherein the structured clinical interview for DSM-IV (SCI-D) was conducted by two trained psychiatrists with more than 5 years of experience; (3) Psychiatric symptoms are assessed on the Positive and Negative Syndrome Scale (PANSS)^21^, which requires a score of over 50 points; (4) Must be experiencing their first episode, have a duration of psychosis of no more than 2 years and have been on antipsychotic medication for no more than 6 months before participating in this study; and (5) Had no history of electroconvulsive therapy (ECT) within 6 months. The exclusion criteria were as follows: (1) other psychotic or affective disorders, mental retardation, drug dependence, organic brain lesions, and/or physical diseases; and (2) contraindications for magnetic resonance imaging (MRI). Thirty-one patients with FSZ met the inclusion criteria to participate in the study, but three patients were excluded after failing to complete the psychophysical experiment. HCs were selected based on the following inclusion criteria: (1) gender-, age- and education-matched to the patient group; and (2) no history of mental illness and no family history of mental illness. Exclusion criteria were the same as the exclusion criteria for the patient group. Finally, 33 HC participants finished our study.

### Behaviour experiment

**Figure 1:**
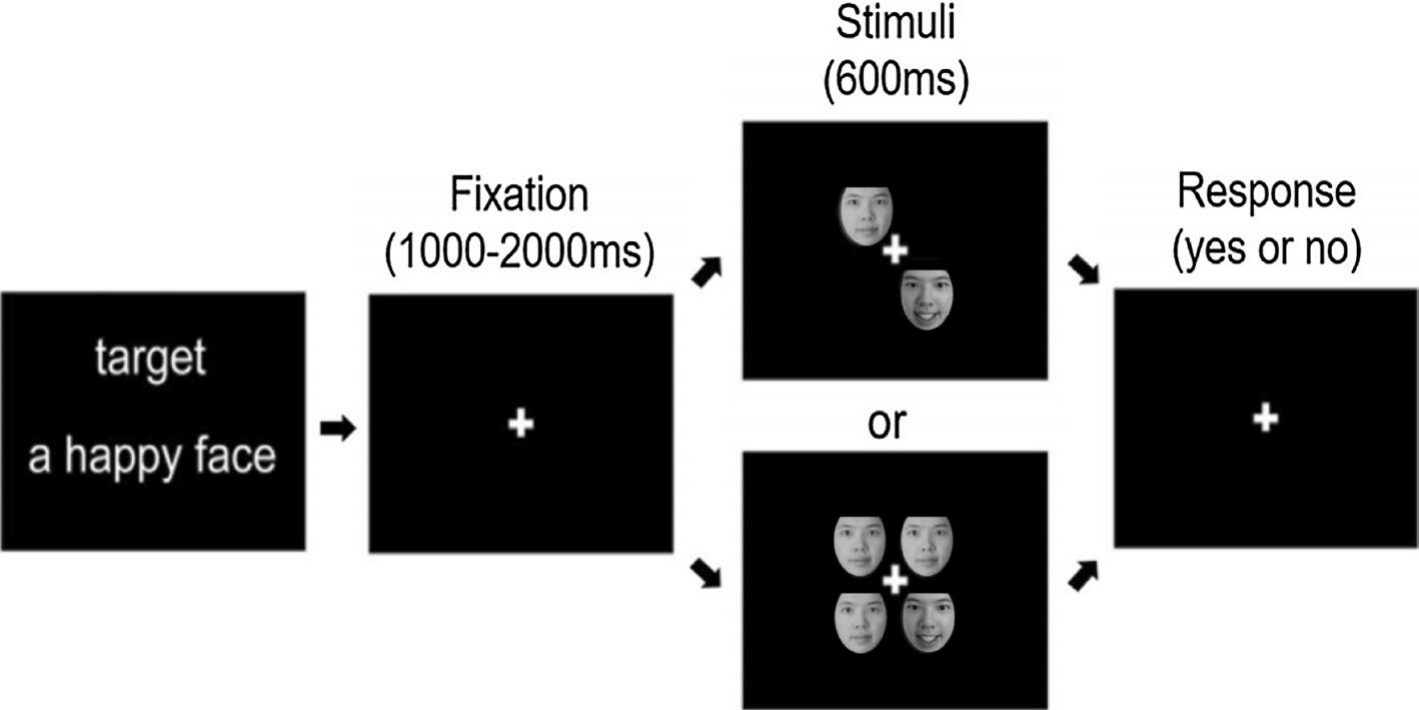
Schematic demonstration of the experimental procedure for happy face search. The face in the picture is from the first author of this study, Qijie Kuang.

Each participant completed two visual search experiments to assess FER ability, and the process is shown in Fig. 1. In the experiments, all subjects were asked to complete the happy FER experiment first and then the fearful FER experiment. The experimental procedure is as follows. Firstly, a small white cross present at the centre of the monitor lasted 1000–2000 ms. Subsequently, two (low-load) or four (high-load) faces are presented for 600 ms, included or excluded emotional faces. Emotional faces are presented in half of the trials. It is worth mentioning that all the figures used during the experiment were selected from the Chinese Facial Affective Picture System (CFAPS)^22^, including thirty-two happy faces, thirty-two fearful faces and sixty-four neutral faces. The hair, ears, and face contour of all faces were excluded via Photoshop. Once faces disappear, subjects need to judge as quickly and accurately as possible ("n" if it contains an emotional face, or "m" if it does not). Next, the experimental procedure begins to cycle and the small white cross present again in the centre of the monitor. Each FER experiment contains six blocks and each block contains 40 trials. Adequate rest periods between two experiments were ensured in order to obtain the best results. Worthy of mention, all subjects were given appropriate instruction and some practice to understand the experiment beforehand.

### Imaging acquisition and processing

Upon completion of the psychophysical experiment, magnetic resonance data collection was performed on the same day. The collection of imaging data was performed in the magnetic resonance chamber of the radiology department by an attending level or higher professional technician from the radiology department. All subjects were scanned with a Philips 3.0 T MRI system, and the signals were received by a standard head coil. During the scan, subjects were asked to secure their heads with supporting foam pads and to minimize the noise with rubber ear buds.

Whole-brain resting state fMRI (rs-fMRI) scans comprising a total of 240 echo-planar imaging (EPI) volumes were obtained with the following parameters: repetition time (TR) = 2000 ms, echo time (TE) = 30 ms, flip angle = 90°, matrix = 64 × 64, slice thickness = 4 mm, slice number = 33, voxel size = 3.44 × 3.44 × 4.60 mm, slice gap, and field of view (FOV) = 192 mm × 192 mm. Rs-fMRI data were analysed and processed with RESTPLUS^23^ in MATLAB 2013b. The preprocessing included the removal of the first ten dummy scans, slice timing correction, realignment, spatial standardization (normalized to Montreal Neurological Institute (MNI) space by using the conventional EPI template), smoothing with a Gaussian kernel of full width-at-half-maximum 8.0 mm, detrending. In addition, white matter, cerebrospinal fluid, and head motion based on Friston 24-parameter model were regressed as the nuisance variables.

To calculate fALFF, the fast Fourier transform (FFT) of the time series of the whole-brain signal was calculated first, converting the signal into a frequency domain power spectrum. Then, the square root of the power spectrum at each frequency was averaged across the filtered band (0.01–0.08 Hz) to obtain the ALFF of the signal. The fALFF was obtained by dividing the ALFF by the full-band amplitude. The fALFF of each voxel was then divided by the global mean of the fALFF to derive mfALFF, which could eliminate differences in whole-brain fALFF at the overall level between individuals. Finally, the mfALFF signal of the ROIs was extracted by the RESTPLUS Toolbox.

High-resolution 3D-T1 structural images were collected following the rs-fMRI scan. The T1 structure images were obtained using a planar imaging sequence of gradient echoes with the following parameters: TR = 8.2 ms, TE = 3.8 ms, flip angle = 7 degrees, matrix = 256 × 256, thickness = 1.0 mm, scanning layers = 165. Voxel-based morphometry (VBM) was employed to explore the GMVs of different brain regions in the two groups. The preprocessing of the 3D-T1MRI was performed with the SPM8 sub-tool (http://www.fil.ion.ucl.ac.uk/spm/software/spm8) and included the following steps. First, 3D-T1 MRI images were segmented into grey matter (GM), white matter (WM) and cerebrospinal fluid. Then, the GM concentration maps were normalized to MNI space. The GMV were obtained by multiplying the GM concentration map by the non-linear determinants obtained from the spatial normalization step. Finally, the GMV were smoothed using a Gaussian kernel of full width-at-half-maximum 6.0 mm. The GMV of the ROIs were extracted by the RESTPLUS Toolbox.

### ROI acquisition

A series of meta-analyses of human fMRI data were downloaded using Neurosynth (www.neurosynth.org^24^) to identify brain regions associated with FER ability. Neurosynth, a platform that automatically synthesizing and analyses the results of many different neuroimaging studies, and a list of search terms are generated after integration and classification of high-frequency words. Neurosynth was used to select the ROIs for our study with search items related to facial emotion. "Emotional faces" and "facial expressions" were selected as keywords for searching the fMRI series. The areas of overlap within both domains using the imaging calculator from SPM 12 (http://www.fil.ion.ucl.ac.uk/spm/software/spm12) were used as ROIs (*p* < 0.01, FDR corrected). BrainNet Viewer^25^ (Version 1.7, https://www.nitrc.org/projects/bnv/) was used to visualize the acquired brain regions.

### Statistical analysis

All values of subjects were indicated by mean (SD), except for gender. Two sample t-tests and chi-square tests were used to compare the basic demographic characteristics between the two groups. Average accuracy and correct reaction time (RT) were considered the behavioural results. The psychophysical experiment was a 2 (stimulus: happy/fearful faces) × 2(condition load: 2 or high-load) × 2 (subject group: schizophrenia or HC) mixed design, in which condition load and stimulation were within-subject factors and subject group (schizophrenia or HC) was a between-subject factor. A multiple t-test with Bonferroni adjustment was performed to compare the differences in accuracy or correct RT between two groups. Additionally, partial correlation analyses with Bonferroni adjustment were employed to explore the correlation between FER ability and mfALFF of the ROIs in order to control for several potential confounders. The confounders included gender, age, education, duration of illness, antipsychotic dose, PANSS total score and GMV of ROIs. All statistical tests were conducted as two-tailed tests with 0.05 as the level of significance (α), while the multiple testing threshold was set at 0.013 (0.05/4). The effect size (ES) of cohen'd method was provided for each statistical test. All statistical analyses were performed using SPSS 22.0.

### Ethical approval

The authors assert that all procedures contributing to this work comply with the ethical standards of the relevant national committees and with the Helsinki Declaration. This study was approved by the Institutional Review Board of the Affiliated Brain Hospital of Guangzhou Medical University, and all subjects provided informed written consent for participation.

## Results

### Demographic and clinical characteristics

A total of twenty-eight patients and thirty-three HC participants finished our study. Age and education of the two groups conformed to the homogeneity of variance principle. There were no significant differences in items of gender (*χ*^2^ = 0.46, *p* = 0.500), age (t = 0.59, *p* = 0.557) or education (t = 0.42, *p* = 0.674) between the two groups. The basic demographic and descriptive characteristics of the participants are shown in Table 1.

**Table 1:** Clinical, demographic characteristics and FER ability of the participants.

	Patients with FSZ (n = 28)	HCs (n = 33)	χ^2^/t	*p*
Gender (male/female)^a^	16/12	16/17	0.46^b^	0.500
Age (years)	25.14 (6.87)	24.24 (5.03)	0.59	0.557
Education (years)	11.32 (3.26)	11.67 (3.11)	0.42	0.674
Duration of illness (months)	8.71 (7.19)	–	–	–
Antipsychotics drug use (mg)^b^	217.38 (230.41)	–	–	–
PANSS positive symptoms	17.36 (5.18)	–	–	–
PANSS negative symptoms	13.61 (4.25)	–	–	–
PANSS psychopathology symptoms	34.14 (8.77)	–	–	–
PANSS total	65.11 (15.48)	–	–	–
Low-load happy stimulus accuracy	0.92 (0.06)	0.96 (0.05)	2.75	0.008^c^
High-load happy stimulus accuracy	0.85 (0.07)	0.91 (0.07)	3.62	0.001^c^
Low-load fearful stimulus accuracy	0.77 (0.12)	0.89 (0.07)	4.70	< .001^c^
High-load fearful stimulus accuracy	0.71 (0.09)	0.83 (0.08)	5.30	< .001^c^
Low-load happy stimulus correct RT	0.42 (0.24)	0.36 (0.25)	0.83	0.408^c^
High-load happy stimulus correct RT	0.57 (0.34)	0.45 (0.23)	1.59	0.116^c^
Low-load fearful stimulus correct RT	0.52 (0.24)	0.42 (0.20)	1.77	0.082^c^
High-load fearful stimulus correct RT	0.60 (0.27)	0.51 (0.24)	1.44	0.157^c^

Values are presented as mean (SD). PANSS, Positive and Negative Syndrome Scale; FSZ, First-episode schizophrenia; HCs, Healthy controls; RT, Reaction time.

^a^Chi-square test; b equivalent to Chlorpromazine dosage; c Significantly different between two groups after Bonferroni adjustments (*p* < 0.05/4).

### Behavioural performance of the visual search task

In the psychophysical experiment, the average accuracy and correct RT were then calculated for each condition (Table 1). Regarding the accuracy results, only the interaction between stimulus and subject group was significant, F = 10.96, *p* = 0.001, ES = 0.044, indicating that the FER accuracy in schizophrenia patients was affected differently by stimuli than in HCs. Significant main effects were observed for condition load, F = 36.64, *p* < 0.001, ES = 0.133, which indicated a lower accuracy for the high-load. Multiple t-tests showed that the accuracy for each condition was significant (happy/low-load: t = 2.75, *p* = 0.008, ES = 0.706; happy/high-load: t = 3.62, *p* = 0.001, ES = 0.931; fearful/low-load: t = 4.70, *p* < 0.001, ES = 1.208, fearful/high-load: t = 5.30, *p* < 0.001, ES = 1.362), indicating that the FER ability of patients with FSZ was impaired compared to the FER ability of the control group.

Regarding the correct RT results, none of the interactions among the above mentioned factors was significant, indicating that the FER correct RT of the two groups were not affected differently by stimulus or load. However, the main effects were significant for load, F = 9.69, *p* = 0.002, ES = 0.039, which indicated that the correct RT was longer for the high-load experiment but was similar for the different stimuli for both loads of subjects. The correct RT of each condition was not significant (happy/low-load: t = 0.83, *p* = 0.408, ES = 0.214; happy/high-load: t = 1.59, *p* = 0.116, ES = 0.410; fearful/low-load: t = 1.77, *p* = 0.082, ES = 0.455; fearful/high-load: t = 1.44, *p* = 0.157, ES = 0.369), which indicated that the correct RT of the patients was similar to the RT of the HCs for each condition.

### ROIs related to facial emotion

**Figure 2:**
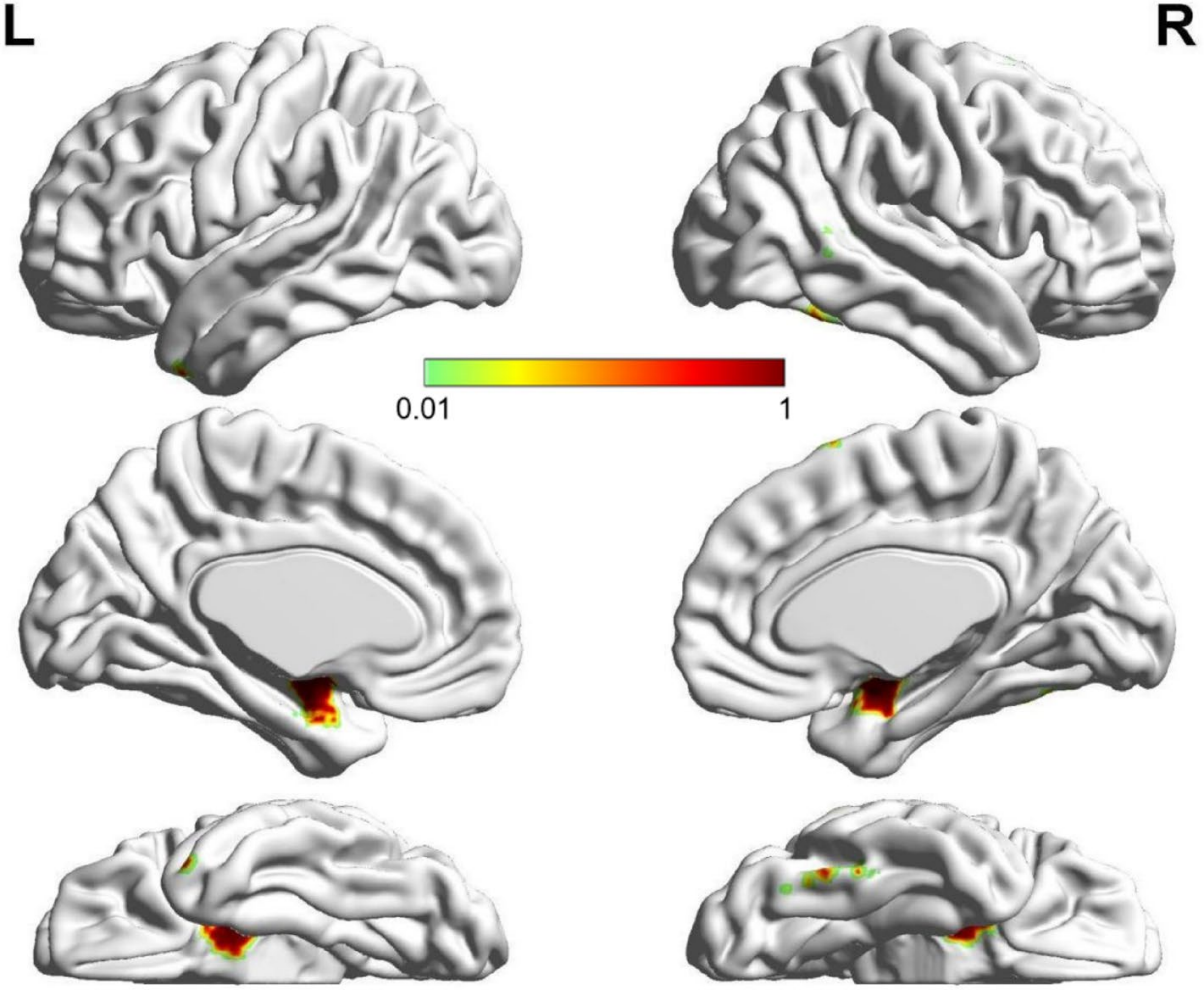
The lateral and medial view of brain regions related to both "emotional faces" and "facial expressions" using Neurosynth (*p* < 0.01, FDR corrected, with a minimum cluster size of 10 voxels). The brain regions associated with FER obtained by using Neurosynth included bilateral AMY, bilateral HIP, right FFG and right SMA. The figure was generated by a brain region visualization tool of "BrainNet Viewer". The color bars showed the correlation between FER ability and brain regions based on meta-analysis. AMY, amygdala; HIP, hippocampus; FFG, fusiform gyrus; SMA, supplementary motor area.

The collection of meta-analysis data using Neurosynth terminated on February 1, 2021. One hundred and 250 studies from the automated meta-analysis survived for the search terms "emotional faces" and "facial expressions", respectively. The brain regions for the search term "emotional faces" included mainly the bilateral AMY, bilateral HIP, and bilateral FFG. The brain regions for the search term "facial expressions" included mainly the bilateral AMY, bilateral HIP, right FFG, left inferior temporal gyrus, right inferior occipital gyrus, right triangular part of the inferior frontal gyrus, right superior temporal gyrus, and right middle temporal gyrus. The ROIs, formed from the area of overlap across the two domains related to facial emotions, included the bilateral AMY, bilateral HIP, right FFG, and right supplementary motor area (SMA). The results are described in detail in Table 2, and an illustration is given in Fig. 2.

**Table 2:** ROIs related to facial emotions.

Brain region (AAL)	Peak MNI coordinates			BA	Cluster size (voxels)
	x	y	z		
Left AMY/Left HIP	− 30	− 6	− 28	–	484
Right AMY/Right HIP	34	− 6	− 28	–	416
Right FFG	42	− 42	24	37	45
Right SMA	6	14	68	–	11
Left AMY/Left HIP	− 30	− 6	− 28	–	484

All brain regions are thresholded at *p* < 0.01, FDR corrected, with a minimum cluster size of 10 voxels. MNI coordinates of ROIs are reported here. AAL, Anatomical Automatic Labeling; MNI, Montreal Neurological Institute; BA, Brodmann area; AMY, amygdala; HIP, hippocampus; FFG, fusiform gyrus; SMA, supplementary motor area.

### Correlation results

Partial correlation analysis showed that the fALFF of the right FFG was associated with high-load fearful FER accuracy (r = − 0.60, *p* = 0.004) and had a correlation trend with low-load fearful FER accuracy (r = − 0.47, *p* = 0.034). The correlation results between the FER accuracy and the mfALFF of ROIs are described in detail in Table 3. However, no significant correlation was found between the correct RT and the mfALFF of ROIs, and the results are described in Table S1.

**Table 3:** Correlation between mfALFF and FER accuracy of patient group.

	Left AMY/Left HIP		Right AMY/Right HIP		Right FFG		Right SMA	
	r	*p*	r	*p*	r	*p*	r	*p*
Low-load happy emotion	0.10	0.665	− 0.09	0.702	0.06	0.808	0.43	0.051
High-load happy emotion	− 0.17	0.456	− 0.20	0.385	0.14	0.554	0.29	0.198
Low-load fearful emotion	0.22	0.329	0.24	0.301	− **0.47**	**0.034**	0.04	0.863
High-load fearful emotion	0.25	0.277	0.25	0.271	− **0.60**	**0.004**^**a**^	0.16	0.503

Significant values are in [bold].

^a^Significantly correlation after Bonferroni adjustments (*p* < 0.05/4).

## Discussion

Facial emotions represent a complex stimulus that conveys a variety of messages that are important for social interaction. To our knowledge, this is the first MRI study to explore the relationship between the fALFF of brain regions related to facial emotion and FER ability in patients with schizophrenia. Furthermore, as previous studies have apparently determined the effect of brain region GMV on FER ability, our study controlled for GMV factors. Our study revealed that patients with FSZ had worse FER accuracy than HCs. In addition, we observed the relationship between FER ability and the fALFF of the right FFG in FSZ patients. Our findings suggest that FER ability is correlated with resting-state intrinsic activity in some brain regions related to facial emotion.

In the behavioural test, we adopted a visual search task to behaviourally measure the FER ability in schizophrenia, which is more concerned with the detection and identification of stimuli compared with the match-to-sample task^26^. Regarding accuracy, similar to the findings of most previous studies, our test showed that patients with FSZ performed worse in FER than HCs^27,28^. Furthermore, only the interaction between stimulus and group was significant, which may be caused by the fact that the accuracy of FER in schizophrenia was impaired more severely for fearful facial emotion than happy facial emotion. Previous studies showing that in schizophrenia, negative FER^29,30^, including fearful emotion, was more severe than positive FER. Similar results were found in FER research of patients with FSZ. Bonfils et al.^31^ showed that patients with FSZ had the highest accuracy in recognizing happy facial emotion and the least accuracy in recognizing fearful facial emotion, which suggested FSZ patients had more difficulty in identifying fearful facial emotion. Regarding the correct RT, our results showed that the difference between the two groups was not significant, although the RTs of the patients with schizophrenia were longer than those of the HCs for each condition. This result was different from the result of a previous study, which indicated that disgusted FER in patients with schizophrenia had a longer RT than the RT in HCs^32^. The reasons for the inconsistent results might contribute to the possibility of differences in stimuli and differences in the tasks used. The main effect consequences for both accuracy and correct RT suggested better performance on the low-load task than on the high-load task, which was similar to the results of another previous study by our group^8^.

The bilateral AMY, bilateral HIP, right FFG, and right SMA were preserved after analysis. Previous MRI findings have shown detailed intricate connection of the FFG with the HIP and AMY, which appear to underpin the network that processes facial emotion^33^. As the core brain region of facial emotion cognition, the function of the AMY is similar in schizophrenia, as indicated in a meta-analysis showing reduced AMY activation in response to facial emotion processing in schizophrenia^34^. Maher et al.^35^ concluded that patients with schizophrenia had significantly lower neural activity by activation in the AMY fearful and happy faces. Similar results were obtained by Wang et al. in schizophrenia patients with high negative symptoms^36^, and they found decreased functional connectivity between the AMY, the ventral medial prefrontal cortex and dorsolateral prefrontal cortex. Additionally, patients with AMY damage showed reduced emotional response in the FFA during the recognition of facial expressions^37^.

A negative correlation was found between altered right FFG fALFF value and fearful FER accuracy in patients with FSZ, which suggested that IBA of the right FFG may correlate with the fearful FER ability in schizophrenia. Although no study on the relationship between fALFF value of FFG and FER ability has been conducted, a large number of studies have shown that the FFG is closely associated with FER^38,39^. The FFG, especially the right FFG, supports face processing, social communication, and facial identity processing^40^. In previous studies of structural MRI, Zhang et al.^28^ found that GMV of the FFG was related to FER in schizophrenia. A similar result was found by Jung et al.^41^, who found that GMV in the FFG was positively correlated with FER and emotional intensity recognition. FMRI research showed functional disconnection between the visual cortex and right FFA in schizophrenia^42^. Some studies hypothesized that the FFA was involved in processing emotional expressions^43^. Multi-voxel pattern-analysis approaches to functional neuroimaging data have shown that emotion categories could be decoded from responses to facial expressions in the FFG^44^. Unfortunately, neither AMY nor HIP was found to be correlated with FER accuracy in the visual search task. Two reasons might cause this result. First, evidence suggests that AMY includes several functional subregions^45^. Our test extracted only the fALFF signal of AMY, which might be a confounding factor for the result. The other reason might be the possibility of differences in the behaviour experiment used.

However, there has been little previous research on FER RT. Some studies have shown that hippocampal brain volume in older adults is positively correlated with the speed of correct FER^46^. Another study including patients with FSZ showed that RT for a high degree of disgust FER task was longer than HCs and increased activation of the left and right middle temporal gyrus and right caudate in response to the high degree of disgust stimulus^32^. However, they did not conduct a correlation analysis to examine the relationship between prolonged RT and activated brain regions. Therefore, the relationship between RT of FER and the intrinsic activity of the resting-state in brain regions needs further investigation.

## Limitations

The comparatively small sample size is a limitation of our study. In addition, all participants were investigated using a cross-sectional design, and the following-up analysis is required. Furthermore, stimuli should be examined in further studies because our test included only fearful faces as negative stimuli. At last, fALFF was used to assess intrinsic brain activity in this study. Other methods, such as ReHo and functional connectivity, should be used in further studies to better understand the underlying neural mechanism between schizophrenia and FER ability.

## Conclusions

In summary, the accuracy of happy and fearful FER in the FSZ patients were found to be impaired in our investigation. Furthermore, our research found that FER ability is correlated with resting-state intrinsic activity in facial emotion-related brain regions, which may provide a reference for the study of FER deficiency in schizophrenia.

## Supplementary Information

Supplementary Information.

## Data Availability

Te data that support the findings of this study are available on request from the corresponding author.
