# Supplementary material for: Association between fractional amplitude of low-frequency fluctuation (fALFF) and facial emotion recognition ability in first-episode schizophrenia patients: a fMRI study
Source: Sci Rep. 2022 Nov 15;12:19561. doi: 10.1038/s41598-022-24258-7 (PMC9666540; doi:10.1038/s41598-022-24258-7)
Supplement: Supplementary file 1 — Supplementary Information. [file 41598_2022_24258_MOESM1_ESM.docx]

Supplementary Table 1. Correlation between mfALFF and FER correct RT of patient group.

|  | Left AMY/  Left HIP | | Right AMY/Right HIP | | Right FFG | | Right SMA | |
| --- | --- | --- | --- | --- | --- | --- | --- | --- |
|  | r | *p* | r | *p* | r | *p* | r | *p* |
| Low-load  happy emotion | 0.11 | 0.628 | 0.29 | 0.200 | -0.11 | 0.625 | -0.20 | 0.397 |
| High-load  happy emotion | 0.10 | 0.664 | 0.15 | 0.523 | -0.22 | 0.329 | -0.02 | 0.948 |
| Low-load  fearful emotion | -0.05 | 0.844 | 0.02 | 0.925 | 0.16 | 0.497 | 0.07 | 0.755 |
| High-load  fearful emotion | -0.05 | 0.841 | -0.02 | 0.919 | 0.15 | 0.524 | -0.03 | 0.894 |
